# Industrial action and emergency department attendances in England, 2022–2024: a retrospective multicentre causal mediation analysis

**DOI:** 10.1177/01410768261461989

**Published:** 2026-08-09

**Authors:** Steven Wyatt, Mohammed A. Mohammed, Jonathan Clarke, Marta Garcia-Fiñana, Iain Buchan

**Affiliations:** 1 The Strategy Unit, Birmingham, England, UK; 2 University of Bradford, Bradford, England, UK; 3 Cardiff Business School, University of Cardiff, Cardiff, Wales, UK; 4 The Health Foundation, London, UK; 5 Institute of Population Health, University of Liverpool, Liverpool, England, UK; 6 University of Liverpool, Liverpool, England, UK

**Keywords:** Health service research, non-clinical, other emergency medicine, emergency medicine, clinical, health policy

## Abstract

**Objectives:**

Between December 2022 and July 2024, the English National Health Service (NHS) experienced 27 periods of industrial action spanning 78 days, involving hospital and ambulance staff. We examined the direct and indirect impacts of these strikes on emergency department (ED) performance

**Design:**

A retrospective causal mediation analysis. Effects were estimated using mixed effects accelerated failure time, linear and generalised linear regression models with patient- and hospital-level covariates within a causal mediation framework.

**Setting:**

20 major (Type 1) EDs in England with high-quality linkage across ED, inpatient, and imaging data.

**Participants:**

Patients attending these EDs between August 2022 and July 2024.

**Main outcome measures:**

A patient’s time in ED analysed in relation to strike activity at the mid-point of attendance by one or more staff groups: (1) resident/junior doctors, (2) consultants, (3) both resident and consultant doctors, (4) paramedics and (5) nurses.

**Results:**

Average duration of ED attendances reduced on days affected by residents’ strikes (-12.6%, 95% CI -10.4% to -14.8%), consultant strikes (-5.6%, 95% CI -2.7% to -8.4%), paramedic strikes (-4.5%, 95% CI -2.0% to -6.8%), but increased when residents and consultants were on strike simultaneously (+7.2%, 95% CI 3.8%–10.7%). There were no significant changes to ED durations during nurse strikes (-1.7%, 95% CI -1.5% to 4.7%). Changes in emergency inpatient occupancy explained a proportion of the change in average ED durations (resident doctor 10.4%, consultants 53.3%, combined doctors 5.4% and paramedics 14.3%). The impact of strikes on ED attendance volumes was mixed. The net effect of these changes explained 1.5%, 16.8% and 28.8% of the change in average ED durations observed during residents, combined doctor and paramedic strikes, respectively. During the residents’ strikes, patients were more likely to receive treatments in ED (adjusted incident risk ratio 1.013, 95% CI 1.008–1.018), but less likely to be admitted (adjusted odds ratio 0.963, 95% CI 0.946–0.980). Efforts to meet the 4-h target intensified during the resident, consultant and combined doctors strike, but reduced during the nurse and paramedic strikes. This explained 2.7% and 17.1% of the changes in average ED durations observed during the resident and consultant strikes, respectively. There were substantial reductions in many forms of planned hospital care during the resident, consultant and combined doctor strikes, but these did not contribute to the observed reductions in ED durations.

**Conclusion:**

Industrial action – particularly by resident and consultant doctors – was associated with shorter ED stays, partly mediated by greater emergency bed availability, lower demand and intensified throughput efforts. However, much of the reduction during resident strikes remained unexplained, suggesting unmeasured operational or behavioural adaptations. Substantial reductions in planned care did not significantly affect ED performance.

## Introduction

Industrial action in the form of a strike has been defined as a ‘temporary stoppage of work by a group of employees in order to express a grievance or enforce a demand’.^1^ Strikes are intended to exert pressure during labour disputes and commonly result in some degree of service disruption. In healthcare, however, the consequences of strike action are especially significant because disruption may affect access to, continuity of and quality of patient care, although these effects are not experienced uniformly across patients, staff groups or different parts of the health system. Moreover, strikes in healthcare raise particular ethical concerns because they sit at the intersection of workers’ rights to collective bargaining and professional obligations to patients. These concerns include delayed or interrupted care, potential harms arising from reduced staffing or altered service provision and questions about how the principles of beneficence and non-maleficence should be balanced against efforts to secure better pay, staffing and working conditions. Nevertheless, healthcare strikes are generally recognised as a lawful and legitimate form of collective bargaining and have occurred in both low- and high-income countries worldwide.^2^ In responding to strike action, unions, healthcare providers and governments must therefore weigh short-term risks and service disruption against the possibility of longer-term improvements in workforce conditions and retention, while ensuring that the public is not exposed to unwarranted harm.

Strike action in the English National Health Service (NHS) has occurred periodically since the late 20th century, including ambulance staff strikes in 1989–1990 and consultants’ industrial action in 2012.^3^ However, the period from December 2022 to July 2024 was exceptional in both scale and significance, representing the most sustained and adversarial phase of industrial relations in the NHS since its founding. The 2022 nursing strikes were the first in the 106-year history of the Royal College of Nursing, highlighting the unprecedented nature of this period. Among resident doctors, 98% of those voting in the British Medical Association ballot supported industrial action, triggering a prolonged dispute. Across the NHS, this wider wave of action comprised 27 strike periods over 78 days. Initially, strikes were led by nurses and paramedics, largely concluding by May 2023, while resident doctors began striking in June 2023 and continued until July 2024. Consultants also undertook several periods of industrial action during mid-2023, at times coinciding with resident doctor strikes. Although the 2015–2016 resident doctors’ dispute had already marked the first full withdrawal of emergency care in NHS history, the 2022–2024 wave of industrial action was unprecedented in its breadth, duration and operational impact.

Whilst some studies have explored the impact of this period of industrial action on planned care and elective waiting lists,^4,5,6^ the implications for the urgent and emergency care system – particularly within Emergency Departments (EDs) – has received comparatively less attention.^7^ This is an important gap because EDs typically operate under considerable pressure from fluctuating and frequently high levels of demand. Under such conditions, patient flow may become constrained, treatment delays may lengthen and overcrowding may occur, all of which might adversely affect patient experience, staff well-being, service efficiency and potentially the safety and quality of care.^8^ However, the relationship between service pressure and patient outcomes is complex and the effects of strike action on ED performance merits careful study. Indeed, it was notable that during the strikes the President of the Royal College of Emergency Medicine stated that ‘in the ED, everything works better than usual’.^3^ One possible explanation is the extensive planning undertaken by hospital trusts and wider health systems to mitigate the impact of strikes on patient care and outcomes. These preparations primarily focus on ensuring adequate clinical cover by non-striking staff groups – for example, consultants, senior nurses and advanced clinical practitioners covering work usually undertaken by resident doctors – but may also include diverting some lower-acuity urgent care patients to alternative services, discharging inpatients who are medically fit in order to free beds and rescheduling some planned procedures and outpatient attendances.

In this study, we sought to determine the extent to which patients’ duration in EDs changed during strike periods and to investigate which changes in patient or staff behaviour, service or operational contingencies on strike days may have influenced patient flow within EDs.

## Methods

### Population, setting and data sources

The analysis relates to first, completed, unplanned attendances at major (Type 1) EDs managed by 20 NHS acute hospital trusts in England between 1 August 2022 and 31 July 2024. Type 1 departments are typically consultant-led, open 24 h a day and have full resuscitation facilities. We excluded attendances relating to patients who were not resident in England (0.4%), or who died in or before arrival at ED (<0.1%)

The 20 NHS hospitals were selected non-randomly, based on the quality and completeness of key fields in the emergency care (ECDS),^9^ admitted patient care (APC)^10^ and diagnostic imaging (DID)^11^ datasets during the study period. Hospitals were included in our analysis if (1) at least 85% of ECDS records had a valid entry in the investigations, chief complaint and acuity fields, (2) at least 98% of APC records had a valid admission and discharge time each month and (3) information about diagnostic imaging activity was recorded in the DID dataset.

We used ECDS, APC and DID, along with data from the Outpatient Attendance dataset (OPA),^5^ the Community Services Dataset (CSDS)^12^ and the GP appointments dataset (GPA)^13^ to derive a dataset with patient, provider and area-level variables.

### Causal theory development

Although the days that NHS staff choose to strike will have been subject to much discussion and negotiation, we consider the date of a strike as being essentially random. We leverage this randomness, along with the very unusual contexts that strike action creates, to generate insight that may be generalised. We developed and refined a set of causal theories to set out the factors that influence a patient’s duration in ED, including industrial action. These theories were informed by descriptive analyses, discussions with stakeholders in Trusts and national agencies and a review of the relevant literature. Our causal theories are documented as directed acyclic graphs (see Supplemental Figure S3).

### Study variables

Four types of variables were constructed (outcome, exposures, pre-exposure covariates and potential mediators), for each attendance at an ED provided by one of our selected NHS Trusts in our study period. We excluded 828,796 (17.4%) records that represented either a follow-up attendance, attendances for patients who lived outside England or were brought in dead or died in the department, and those without a valid attendance category, arrival mode, sex or age.

Our primary outcome variable was the patient’s duration in the ED from arrival to departure or admission. This was derived as the difference in minutes between the arrival and departure time. Two secondary outcomes were also defined. These were binary variables indicating whether the patient’s duration in ED exceeded 4 and 12 h.

We created 5 exposure variables. These were binary variables indicating whether a strike by one or more of the following staff groups was in force at the mid-point of the patients stay in ED: (1) resident doctors, (2) consultants, (3) both resident doctors and consultants, (4) paramedics and (5) nurses. Information about the timings of strikes were taken from the industrial action situation reports, NHS England and relevant Trade Union communications.^14^

There were 11 pre-exposure covariates, which we posited were likely to influence the patient’s duration but were not a function of the operations of ED. These were (1) the patient’s age in years, (2) sex, (3) deprivation decile, (4) acuity, (5) arrival mode, (6) chief complaint group, (7) hour of day, (8) weekday/weekend, (9) winter/other seasons, (10) year and (11) bank holiday. Provider was included as clustering variable to capture context. Pre-exposure covariates 1, 2, 4, 5 and 6 and provider were taken directly from the ECDS dataset. The acuity covariate takes one of five levels: immediate resuscitation, very urgent, urgent, standard and not urgent. Arrival mode was a binary variable indicating whether a patient was conveyed by ambulance. The chief complaint group took 1 of 15 levels: airway/breathing, circulation/chest, drug and alcohol, environmental, eye, gastrointestinal, general, genitourinary, head and neck, injury/musculoskeletal, neurological, obstetrics/gynaecology, psychosocial/behaviour change and skin. Deprivation was derived by linking the patient’s lower super output area of residence in the ECDS dataset to the 2019 Indices of Multiple Deprivation.^15^ The temporal covariates were measured at the mid-point between the patient’s arrival and departure time in the ECDS dataset. Winter was defined as the 4-month period from December to March.

We created 21 potential mediator variables. These include: the total number of (1) ambulance-conveyed and (2) walk-in attendances handled by the patient’s provider on the day of their attendance, the total number of (3) attendances at minor injuries units, (4) contacts with community services, (5) pre-booked and (6) same-day GP appointments in the area of residence the patient lived, on the day of their attendance, the number of (7) elective and (8) non-elective patients in hospital beds in the patient’s provider in the hour closest to the mid-point of their attendance, the number of (9) outpatient attendances. (10) outpatient procedures, (11) non-ED CT scans, (12) non-ED MRI scans, (13) non-ED ultrasound scans and (14) non-ED X-ray scans carried out by the patient’s provider on the day of their attendance, the number of (15) imaging tests, (16) haematological tests, (17) biochemistry tests and (18) other tests performed on the patient during their ED attendance, (19) the number of treatments carried out on the patient during their ED attendance, (20) whether the patient was admitted to hospital at the end of their attendance and (21) the degree of distortion in attendance durations associated with the 4-h ED duration target, at the patient’s provider on the day of their attendance. Mediators 1–3 and 15–21 were derived from the ECDS dataset, mediator 4 from the CSDS, mediators 5 and 6 from the Appointments in GPA, mediators 7 and 8 from the APC dataset, mediators 9 and 10 from the OPA and mediators 11–14 from the imaging/DID dataset. Where the patient’s area of residence was used to determine a mediator variable (i.e. for mediators 3–6), the area is the Integrated Care Board sub-location derived from the patient’s lower super output area of residence at the time of attendance. The mediators that relate to GP appointments (mediators 5 and 6) include those that were booked, but the patient did not attend. Mediators 1–6 and 7–14 were scaled by dividing the daily or hourly rate for the provider or area by the mean rate for that provider or area, and day or hour. To calculate mediators 8 and 9, the number of elective and non-elective patients in hospital beds, we required information on the admission and discharge time of all hospital spells. In the small proportion of cases (0.3%) where one or both of these variables was missing or invalid, we imputed a value (see Supplemental Note S1 for details). Note that these mediators measure the number of patients in beds. Patients are assigned as either elective or non-elective according to their admission method, rather than the bed designation. The 4-h target distortion mediator (21) was calculated as the proportion of attendances with durations between 231 and 250 min (i.e. 10 min before and after 4 h) that were admitted or discharged before 241 min).

### Statistical analysis

We start by describing the frequency and distribution of the ED attendances in terms of the study outcome, the patient and attendance characteristics, and the potential mediators in the presence of the study exposure. In addition, we compare the characteristics of attendances at providers within and outside of our study population.

We develop a regression model that explores how the primary study outcome, duration in ED, varied as a function of our 5 exposure and 11 pre-exposure covariates. This took the form of an accelerated failure time model, with a log-logistic distribution, clustered on provider. We created two supplementary models with our secondary outcome variables, ED duration > 4 h and > 12 h. These were mixed effects logistic regression models with the same exposure and pre-exposure covariates including provider as a random effect.

In some rare cases, a patient’s duration in ED is recorded as 0 min. Since the link-function in the model precludes the outcome taking a zero-value, we added 1 min to all ED durations. Durations were truncated at 4320 min (3 days) in 0.04% of cases. Records with missing values for one or more of the outcome, exposure, pre-treatment and mediator variables were excluded at this stage. To manage model creation times and given the very large number of records in our dataset, the model was built using a 50% random sample of records (see Supplemental Figure S1). These conventions were followed for all subsequent analyses.

Having determined whether each of the strikes led to changes in ED durations, we use causal mediation analysis to examine whether, and the extent to which, this effect could be explained by changes in each of the 21 mediators (see Supplemental Figure S5 for a simplified overview of the causal model). To demonstrate this in line with causal mediation theories, we must show that (1) strikes led to changes in the mediator, and (2) the mediator influenced ED duration having controlled for the presence/absence of strikes. We achieve this by developing a series of exposure–mediator models for (1) and mediator–outcome models for (2).

The exposure–mediator models describe how a mediator varies as a function of the 5 exposure and 11 pre-exposure covariates. These models took the form of mixed effects linear or generalised linear models, with the provider variable treated as a random effect. One model was created for each mediator. Linear models were created when the mediator was a continuous variable (mediators 1–14 and 21). When the mediator was a count (mediators 15–19) or binary (mediator 20), then we created generalised linear models with Poisson or binomial distributions, respectively.

The mediator–outcome models described how ED durations varied as a function of the 5 exposure, 11 pre-exposure covariates and 1 of the mediator variables. One model was created for each mediator. These took the form of accelerated failure time models, with a log-logistic distribution, clustered on provider.

Having developed our exposure–mediator and mediator–outcome models, we assess the extent of causal mediation using the methods developed by Imai et al. and operationalised by Tingley et al.^16,17^ We used the default quasi-Bayesian approximation approach for confidence intervals with 200 Monte-Carlo draws.

We carried out three sensitivity analyses. Firstly, we tested the sensitivity of our results to violation of the sequential ignorability assumption. This assumption states that unobserved confounders between exposure and mediator and between the mediator and outcome can be safely ignored. We tested four selected combinations of exposure and mediator: (i) resident doctor strike and non-elective bed occupancy, (ii) consultant strike and the number of biochemistry tests, (iii) resident doctor and consultant strike and 4-h distortion effect and (iv) paramedics strike and the number of ambulances conveyed ED attendances. Selecting a small number of cases was necessary here because these tests require considerable computing resources. The cases were selected to provide coverage of both exposure and mediators where the mediating effect in the main analysis was strong. The sensitivity analysis was run on a random sample of 200,000 records from our model dataset. Secondly, we assess the extent to which the results change if we allow for causal relationships between three selected pairs of mediators which were plausibly causally related ((i) the number of walk-in ED attendances and ambulance-conveyed attendances, (ii) whether the patient is admitted and non-elective bed occupancy and (iii) the number of imaging tests a patient receives and whether the patient is admitted). This second sensitivity analysis is performed for the resident doctor strike only. For both sensitivity analyses, current methodological limitations required us to deploy simpler model forms. All relationships between exposure and mediator and between mediator and outcome were modelled using simple linear regression and without random effects. Finally, to address concerns about inconsistent practices of recording multiple instances of tests in ED, we assessed whether the main results would be altered if we treated the ED test mediators as binary rather than as count variables.

All analysis was carried out using R version 4.4.3 and making extensive use of the tidyverse, lme4, survival and mediation packages.^17–21^ The analysis code is available on GitHub (https://github.com/The-Strategy-Unit/impact_of_strikes_on_ed_durations). We present our analysis according to the RECORD reporting guidelines.

## Results

### Characteristics of the industrial action

Nurse strikes commenced in December 2022 and ended in May 2023. There were six distinct periods of industrial action, each lasting between 1 and 3 days, and covering 12 days in total. There were six single-day instances of industrial action by paramedics that took place between December 2022 and February 2023. Resident doctor strikes were more numerous and intensive than those of other staff groups (11 periods covering 54 days) and ran for a longer period (March 2023 to July 2024). There were four consultant strikes that took place between June and October 2023, each lasting 2 or 3 days. There were two periods, covering 4 days in September and October 2023 when both resident doctors and consultants were on strike simultaneously.

### Characteristics of study population

Our study dataset, as shown in Table 1 comprised 3,946,696 ED attendances, 15.2% of all such attendances in England over the 2-year study period (between August 2022 and July 2024). These attendances took place at 20 (15.7%) NHS Hospital Trusts with Type 1 EDs. Although NHS Trusts were selected non-randomly based on the quality and completeness of their data, the profile of ED attendances at these Trusts was broadly representative of all such attendances in England during the study period in terms of sex, age, acuity and ED duration. Ambulance-conveyed attendances and patients living in the least deprived areas were slightly over-represented (see Supplemental Table S1).

**Table 1.** ED attendances by patient and presentations characteristics and exposure to industrial action between August 2022 and July 2024.

	Industrial action					
Measure	None	Residents	Consultants	Residents + Consultants	Paramedics	Nurses
# Attendances	3,602,911	227,298	27,823	11,701	31,214	43,733
% of attendances	91.3%	5.8%	0.7%	0.3%	0.8%	1.1%
Mean ED duration (min)	377	323	332	371	390	392
Female %	51.5%	51.7%	51.4%	51.1%	51.4%	51.6%
Mean age (years)	44.1	44.6	45.0	43.3	43.8	43.1
Most deprived decile %	12.7%	12.8%	12.7%	12.4%	13.1%	12.8%
Ambulance conveyed %	31.0%	31.7%	30.1%	28.7%	31.1%	29.9%
Urgent %	53.7%	53.8%	50.4%	51.5%	52.7%	51.8%
Airway/breathing %	9.7%	9.2%	7.1%	9.8%	11.6%	10.8%
Circulation/chest %	11.9%	12.0%	12.1%	12.5%	12.4%	11.7%
Environmental %	0.8%	0.8%	0.7%	0.7%	0.7%	0.8%
Eye %	1.3%	1.3%	1.4%	1.2%	1.3%	1.2%
Gastrointestinal %	12.4%	12.5%	12.6%	11.7%	12.1%	11.8%
General %	10.2%	10.0%	9.5%	9.4%	11.5%	12.2%
Genitourinary %	3.4%	3.4%	3.5%	3.2%	3.0%	3.2%
Head and neck %	4.2%	3.9%	3.3%	4.0%	4.5%	4.8%
Neurological %	8.2%	8.3%	8.5%	8.0%	8.1%	7.8%
Obs/gynaecology %	1.2%	1.2%	1.2%	1.2%	1.1%	1.2%
Psychosocial %	2.0%	2.1%	2.0%	1.8%	2.0%	1.9%
Skin %	5.1%	5.2%	5.8%	5.3%	4.6%	4.9%
Trauma/MSk %	23.6%	23.9%	26.4%	25.6%	21.4%	22.2%
Other complaint %	6.0%	6.2%	6.0%	5.5%	5.4%	5.4%
Out of hours* %	36.5%	36.1%	35.0%	34.3%	37.1%	37.5%
Weekend %	28.4%	24.7%	8.3%	0.0%	0.0%	2.2%
Winter %	31.6%	40.1%	0.0%	0.0%	100.0%	85.8%
Bank holiday %	2.4%	0.0%	0.0%	0.0%	0.0%	12.0%

* Before 8 am or after 8 pm.

The data reveals that the vast majority of ED attendances (91.3%) occurred during periods without industrial action, with significantly fewer attendances during strikes, particularly those involving consultants or both residents and consultants. Mean ED duration was shortest during resident doctor strikes (323 min) and longest during nurse strikes (392 min). Patient demographics – such as age, sex and deprivation levels – remained broadly consistent across all periods. However, variations emerged in clinical and operational patterns: trauma and musculoskeletal issues peaked during consultant strikes (26.4%), while airway/breathing complaints were more common during paramedic (11.6%) and nurse (10.8%) strikes.

Table 2 shows the ED attendances by potential mediating factors and exposure to industrial action. The results show that ambulance arrivals remained stable (~1.00) except for a decrease during paramedic (0.95) and nurse strikes (0.96). Walk-ins were slightly higher during combined doctor strikes (1.08) and lower during paramedic (0.95) and nurse (0.97) strikes. Minor Injury Unit (MIU) attendances dropped notably during paramedic (0.91) and nurse (0.86) strikes. Community contacts were higher during doctor-related strikes, especially resident doctors + consultants (1.13). Pre-booked GP appointments rose sharply during residents + consultants strike (1.25) but fell during consultant (0.94) and nurse (0.91) strikes. Outpatient and elective activity dropped across nearly all strike types – especially during consultant and nurse strikes. Elective inpatients were lowest during residents + consultants (0.82). Outpatient procedures dropped most during nurse strikes (0.82). Non-ED imaging (CT, MRI, Ultrasound, X-ray) decreased during consultant and nurse strikes. X-ray usage was especially low during consultant (0.93), combined doctor (0.93) and nurse (0.92) strikes. In ED, imaging tests per patient and biochemical tests per patient were generally stable, with slightly higher biochemical testing during consultant strikes (2.05). Treatments per patient peaked during resident doctor strikes (1.95). Admission rates dropped to lowest during combined doctor strikes (29.6%) and were highest during paramedic strikes (32.7%). The 4-h performance distortion was lowest during paramedic (0.74) and nurse (0.75) strikes.

**Table 2.** ED attendances by potential mediating factors and exposure to industrial action between August 2022 and July 2024.

	Industrial action					
Potential mediator	None	Residents	Consultants	Residents + Consultants	Paramedics	Nurses
Mean ED ambulance arrivals*	1.00	1.02	1.00	1.02	0.95	0.96
Mean ED walk-ins*	1.02	1.00	1.01	1.08	0.95	0.97
Mean MIU attendances*	1.00	1.00	1.00	1.02	0.91	0.86
Mean community contacts*	1.04	1.11	1.04	1.13	0.94	0.88
Mean pre-booked GP appts*	1.05	0.95	0.94	1.25	1.03	0.91
Mean same-day GP appts*	1.03	1.01	0.93	1.04	1.07	0.96
Mean non-elective inpatients*	1.01	0.99	0.96	0.99	1.02	1.01
Mean elective inpatients*	1.02	0.85	0.84	0.82	0.98	0.92
Mean outpatient attendances*	1.01	0.94	0.89	0.95	1.03	0.88
Mean outpatient procedures*	1.03	1.02	0.96	0.99	0.99	0.82
Mean non-ED CT scans*	1.00	1.00	0.96	0.93	1.04	0.95
Mean non-ED MRI scans*	1.00	1.01	0.95	0.96	1.06	0.96
Mean non-ED ultrasound *scans	1.00	1.02	0.92	0.96	1.07	0.91
Mean non-ED X-rays*	1.00	0.97	0.93	0.93	1.10	0.92
Mean imaging tests/patient	0.61	0.61	0.63	0.61	0.61	0.61
Mean blood tests/patient	0.96	0.96	0.97	0.93	1.01	0.98
Mean biochem. tests/patient	2.01	2.02	2.05	2.00	2.04	1.97
Mean other tests/patient	0.42	0.42	0.41	0.42	0.45	0.43
Mean treatments/patient	1.91	1.95	1.90	1.94	1.93	1.93
% Admitted	31.8%	31.3%	30.8%	29.6%	32.7%	31.6%
Mean 4-h distortion	0.79	0.80	0.81	0.81	0.74	0.75

ED: emergency department.

For mediators shown with an asterisk (*), the value indicates the level of activity when the patient attended ED relative to the average for the day of week or hour for that provider or ICB. See methods for further information. The impact of industrial action on attendance durations.

Our model dataset comprised of 1,684,616 records: a 50% random sample of our study dataset having removed 577,464 (14.6%) records due to missing values. Our model dataset was broadly representative of all attendances at Type 1 EDs in England with respect of patient age, sex, deprivation, arrival mode, acuity and ED durations (see Supplemental Table S1).

**Figure 1.**
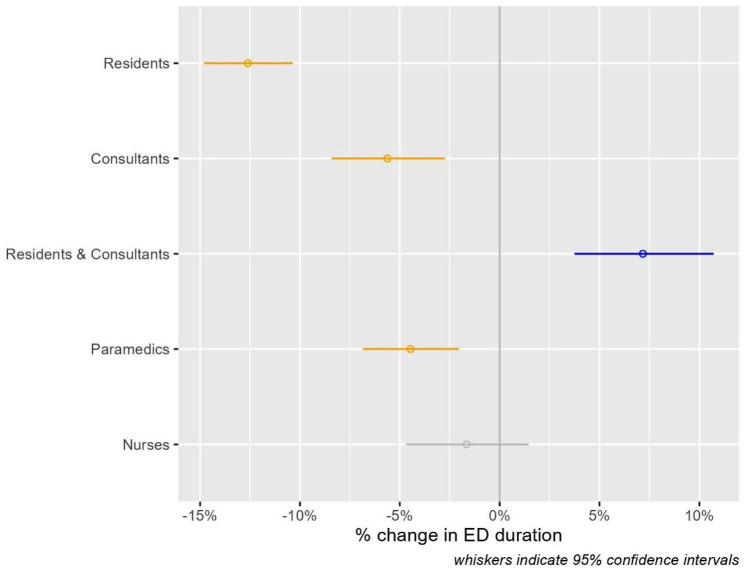
Adjusted change in ED durations for attendances occurring during industrial action, England, between August 2022 and July 2024. ED: emergency department.

Having controlled for case-mix, temporal and provider factors, the time that patients spent in ED reduced by 12.6% (95% CI 10.4%–14.8%) during the resident doctor strikes, 5.6% (95% CI 2.7%–8.4%) during consultant strikes, 4.5% (95% CI 2.0%–6.8%) during paramedic strikes and by 1.7% (95% CI −1.5% to 4.7%) during nurse strikes. When both resident doctors and consultants were on strike, the adjusted duration in ED increased by 7.2% (95% CI 3.8%–10.7%; see Figure 1).

The mean marginal effects of strikes on the duration of ED attendances in our model dataset was −37.9 min (IQR −47.2 to −23.6) during resident doctor strikes, −16.8 min (IQR −20.9 to −10.4) during consultant strikes, −13.3 min (IQR −16.6 to −8.3) during paramedic strikes, −4.9 minutes (IQR −6.1 to −3.1) during nurse strikes and +21.4 min (IQR +13.3 to +26.6) when both residents and consultants were on strike.

The odds of a patient’s duration in ED exceeding 4 h decreased significantly by 37.0% (95% CI 35.0–38.9) during the resident doctors’ strike, by 20.2% (95% CI 12.9%–26.9) during the consultant strike and 12.0% (95% CI 6.2–17.4) during the paramedic strike. Meanwhile, the odds of a 4-h breach increased when both residents and consultants were on strike by 15.6% (95% CI 2.6–30.1). The impact of nurse strikes was modest (0.8% increased odds) and not statistically significant (95% CI −4.8 to +6.7). Similar effects were found in relation to the odds of a 12-h breach, although in this case the nurses strikes were associated with a statistically significant reduction of 8.8% (95% CI 5.5–12.1) in the odds of a breach (see Supplemental Table S2).

### Change in potential mediators during strikes

**Figure 2.**
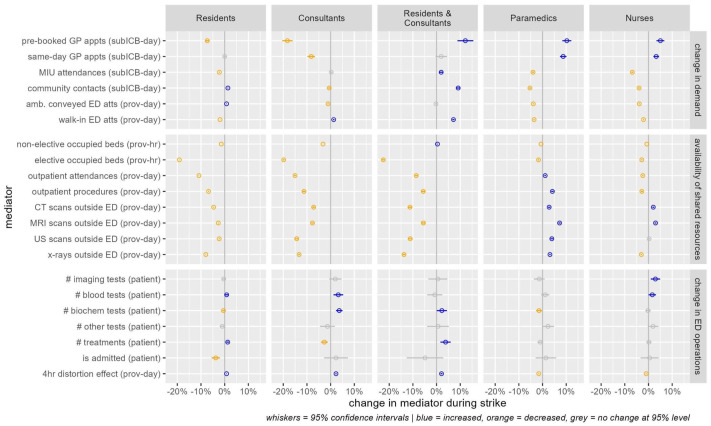
Adjusted change in potential mediators during strikes between August 2022 and July 2024.

Figure 2 display the changes in each of the 21 potential mediators, during each of the 5 strike groups. These are the coefficients of the exposure variables in the mediator models.

Strikes by resident doctors led to significant disruptions across various aspects of healthcare delivery. There was a notable decrease in pre-booked GP appointments, walk-in ED attendances and MIU attendances. Meanwhile ambulance-conveyed ED attendances and community service contacts increased. The availability of shared hospital resources was also impacted, with marked reductions in outpatient attendances, outpatient procedures and diagnostic imaging services such as CT and MRI scans outside ED. There was a reduction in the number of beds occupied by elective and non-elective patients. In EDs, patients were less likely to be admitted (adjusted odds ratio 0.963, 95% CI 0.946–0.980), and to receive a biochemistry test, but slightly more likely to receive a blood test and a treatment (adjusted incident risk ratio 1.013, 95% CI 1.008–1.018). Efforts to achieve the 4-h target increased.

Consultants’ strikes also led to changes. GP appointments reduced, along with community contacts and ambulance-conveyed ED attendances, while walk-in ED attendances increased. Changes in hospital activities outside ED mirrored those that occurred during resident doctors’ strikes. In ED, patients were more likely to receive a blood and biochemistry test, but less likely to receive a treatment. Efforts to achieve the 4-h target increased.

When both residents and consultants went on strike simultaneously, there were increases in pre-booked GP appointments, MIU attendances, community contacts and walk-in ED attendances. Levels of elective bed occupancy, outpatient activity and imaging outside ED reduced, but non-elective bed occupancy increased. In ED, patients were more likely to receive a biochemistry test and a treatment. Efforts to achieve the 4-h target increased.

The changes during paramedic strikes were notably different. GP appointments increased, but community contacts, ED and MIU attendances all decreased. Bed occupancy reduced but there were increases in the level of outpatient and imaging activity outside ED. Patients in ED were less likely to receive a biochemistry test. Efforts to achieve the 4-h target reduced.

The changes during nurse strikes were in many ways similar to those seen during paramedic strikes. GP appointments increased, but community contacts, ED and MIU attendances decreased. Along with reductions in bed occupancy, there were reductions in outpatient activity and X-rays outside ED but increases in CT and MRI scans. In ED, patients were more likely to receive an imaging and blood test. Efforts to achieve the 4-h target reduced.

### The impact of potential mediators on ED durations

**Figure 3.**
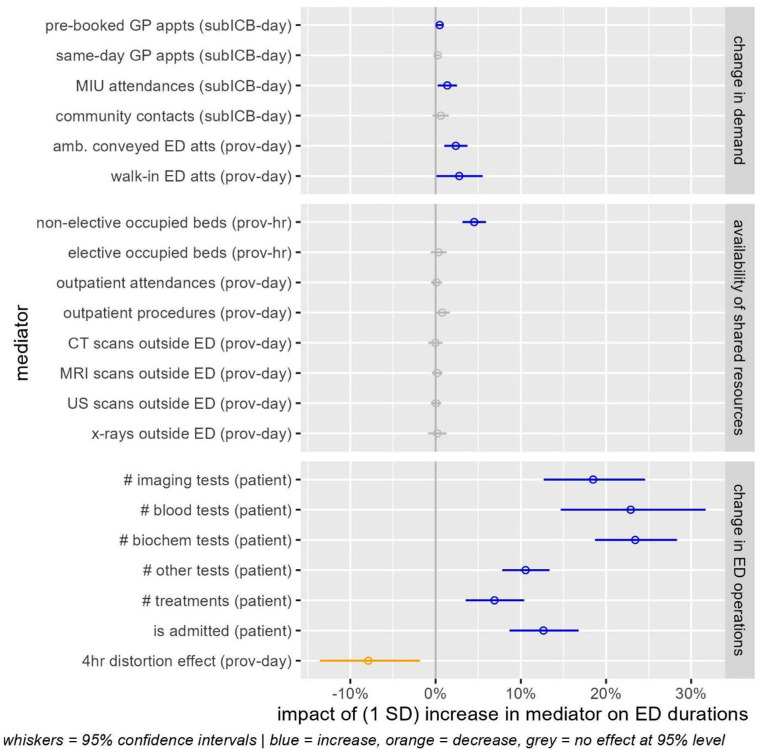
Adjusted impact of change (1 standard deviation) in potential mediators on ED durations between August 2022 and July 2024. ED: emergency department.

Figure 3 shows the average adjusted impact of mediators on ED durations on strike and non-strike days. These are derived from the coefficients of the mediators in the outcome models. In ED, the number of imaging tests, blood tests, biochemistry tests, other tests, treatments and admissions per patient was associated with significant increases in ED durations. In contrast, greater efforts to achieve the 4-h target was linked to a reduction in ED durations. Higher demand for ED and MIU services and higher levels of non-elective bed occupancy were associated with longer ED durations. When pre-booked GP appointments increased, so too did ED durations.

### Proportion of change in ED durations during strikes that was mediated

Table 3 (and Supplemental Figure S2) shows the total effects of strike action on ED durations (reported earlier), and the component of these effects that were brought about by changes in the potential mediators. Mediated effects were generally small in magnitude and often not statistically significant.

**Table 3.** Direct and mediated effect of industrial action on ED durations in minutes with 95% confidence intervals between August 2022 and July 2024.

Effect of industrial action on ED durations (mins)	Residents		Consultants		Residents + Consultants		Paramedics		Nurses	
Total effect	−37.9		−16.8		+21.4		−13.3		−4.9	
Mediated effects										
Change in demand										
Pre-booked GP appointments	−0.1	(−0.2 to 0.0)	−0.2	(−0.7 to −0.1)	+0.2	(0.3 to 0.0)	+0.1	(0 to 0.2)	+0.1	(−0.4 to 0.6)
Same-day GP appointments	0.0	(0.0 to 0.0)	−0.1	(−0.2 to 0.0)	0.0	(0.1 to 0.0)	+0.1	(0 to 0.2)	0.0	(−0.2 to 0.3)
Community service contacts	−0.3	(−0.7 to 0.0)	0.0	(0.0 to 0.1)	+0.3	(0.6 to 0.0)	−0.6	(−1.7 to −0.1)	−0.6	(−8 to 15.4)
MIU/WIC attendances	+0.1	(0.0 to 0.2)	0.0	(−0.1 to 0.0)	+0.6	(2.2 to −0.2)	−0.3	(−1.1 to 0.2)	−0.1	(−2.2 to 2.3)
Amb-conveyed ED attendances	0.4	(0.2 to 0.7)	−0.5	(−1.2 to −0.2)	−0.2	(0.1 to −0.5)	−2.0	(−5.9 to −1)	−1.6	(−15.1 to 20.9)
Walk-in ED attendances	−1.0	(−2.0 to 0.0)	0.7	(0.2 to 1.4)	+3.8	(7.3 to 0.1)	−1.8	(−4.4 to −0.1)	−0.7	(−8.6 to 9.0)
Availability of shared resources										
Non-elective bed occupancy	−3.9	(−5.4 to −2.8)	−8.9	(−19.4 to −5.3)	+1.2	(2.7 to 0.6)	−1.9	(−3.3 to −1.2)	−1.6	(−18.2 to 28.9)
Elective bed occupancy	−0.9	(−3.4 to 1.0)	−1.0	(−4.0 to 1.4)	−1.2	(3.4 to −3.7)	−0.1	(−0.3 to 0.1)	−0.1	(−2.2 to 0.6)
Outpatient attendances	−0.1	(−1.0 to 0.8)	−0.2	(−1.4 to 1.3)	−0.1	(0.5 to −0.8)	0.0	(−0.1 to 0.1)	0.0	(−0.5 to 0.5)
Outpatient procedures	−0.5	(−1.0 to 0.1)	−0.8	(−1.5 to 0.3)	−0.3	(0.1 to −1.2)	+0.3	(−0.1 to 0.9)	−0.1	(−1.3 to 2.2)
Non-ED CT scans	0.0	(−0.4 to 0.4)	0.1	(−0.6 to 0.8)	+0.1	(1.0 to −1.4)	0.0	(−0.2 to 0.5)	0.0	(−0.8 to 0.7)
Non-ED MRI scans	0.0	(−0.2 to 0.1)	−0.2	(−1.3 to 0.3)	−0.1	(0.3 to −0.6)	+0.1	(−0.4 to 0.7)	0.0	(−0.7 to 1.1)
Non-ED Ultra-sound scans	0.0	(−0.1 to 0.1)	−0.1	(−0.9 to 0.8)	−0.1	(0.5 to −0.7)	0.0	(−0.2 to 0.2)	0.0	(−0.1 to 0.1)
Non-ED X-ray scans	−0.2	(−1.2 to 0.9)	−0.2	(−2.7 to 1.8)	−0.4	(1.9 to −2.6)	0.0	(−0.4 to 0.5)	−0.1	(−2.2 to 1.4)
Change in ED operations										
# Imaging tests	−0.2	(−0.6 to 0.2)	+0.9	(−0.4 to 2.7)	+0.2	(2.3 to −2.2)	−0.6	(−1.9 to 0.4)	+0.9	(−8.3 to 22.3)
# Blood tests	+0.6	(0.1 to 1.5)	+2.6	(0.6 to 6.9)	−0.6	(1.6 to −3.9)	+0.8	(−0.3 to 3.6)	+0.8	(−9.8 to 22.7)
# Biochemistry tests	−0.3	(−0.8 to 0.0)	+3.0	(1.6 to 7.3)	1.6	(3.9 to 0.1)	−1.0	(−2.4 to −0.3)	−0.1	(−4.5 to 1.4)
# Other tests	−0.2	(−0.5 to 0.1)	−0.3	(−1.3 to 0.5)	+0.2	(1.5 to −1.0)	+0.5	(−0.1 to 1.6)	+0.3	(−5.4 to 4.1)
# Treatments	0.3	(0.1 to 0.6)	−0.6	(−1.6 to −0.3)	0.9	(2.3 to 0.3)	−0.2	(−0.6 to 0.1)	0.0	(−1.1 to 1.4)
Is admitted	−0.5	(−0.9 to −0.2)	0.3	(−0.2 to 1.3)	−0.7	(0.2 to −3.9)	+0.2	(−0.3 to 1.1)	0.0	(−1.8 to 2.1)
4-h distortion	−1.0	(−1.8 to −0.2)	−2.9	(−10.9 to −0.7)	−2.8	(−0.6 to −8.0)	+1.8	(0.3 to 4.2)	+0.9	(−30.9 to 7.4)

ED: emergency department.

During the resident doctor strikes, a proportion of the reduction in ED durations can be explained by reductions in non-elective bed occupancy, increases in admission thresholds through ED, increased efforts to achieve the 4-h target, reductions in walk-in ED attendances, community service contacts and pre-booked GP appointments. The reduction in ED durations occurred despite some countervailing effects: increases in the number of blood tests and treatments per patient in ED, and an increase in the number of ambulance-conveyed ED attendances.

During consultant strikes, reductions in ED durations were enabled by reductions in non-elective bed occupancy, increased efforts to achieve the 4-h target, a reduction in the number of treatments per patient, the number of ambulance-conveyed ED attendances and pre-booked GP appointments and despite increases in the number of walk-in ED attendances, blood and biochemistry tests per patient in ED.

When residents and consultants were on strike simultaneously, increases in average ED durations were driven in part by increases in walk-in ED attendances, non-elective bed occupancy, the number of biochemistry tests and treatments per patient in ED, community contacts and pre-booked GP appointments and offset somewhat by increased efforts to achieve the 4-h ED target and reductions in ambulance-conveyed ED attendances.

Reductions in average ED durations during the paramedic strikes were facilitated by reductions in ED attendances, non-elective bed occupancy, community contacts and the number of biochemistry tests per patient in ED, despite reduced efforts to achieve the 4-h ED target and reductions in GP appointments.

No significant mediated effects were identified to explain the non-significant reduction in ED durations during nurse strikes.

Notably, the substantial reductions in planned hospital activities (elective bed occupancy, outpatient and imaging activity outside ED) did *not* mediate the changes in ED durations during strikes.

### Sensitivity analyses

The first sensitivity analysis tested the extent to which the mediated effects described above are sensitive to violations of the sequential ignorability assumption. The low values of the sensitivity parameter rho at which the average causal mediation is zero suggest that the mediation effect is highly sensitive to unobserved confounding. If, therefore, pre- or post-treatment confounders exist, then the mediated effects described above may be unreliable. (see Supplemental Table S3). In ‘Discussion’ section, we offer a view about the conceptual plausibility of such confounders.

The second sensitivity analysis explores the impact of causal relationships between mediators. We selected three pairs of mediators where the a priori expectation of a causal relationship was strong. In two cases, these causal effects did not substantially impact the average causal mediated effects (see Supplemental Figure S3). In one case, taking account of the causal relationship between mediators increased the scale of the estimated mediated effect. It is worth noting, however, that these tests were necessarily performed using model forms that were considerably simpler than in our main analysis. It is unclear if these effects would remain if we were able to test for causal interactions between mediators within our main modelling paradigm.

The third sensitivity analysis assessed the relevance of the construction of ED diagnostic testing mediators, and in particular, whether treating these as binary rather than count variables would alter the main results narrative. In most cases, the results for this sensitivity analysis were similar to those of the main analysis. In a small number of cases, significant results in the main analysis became non-significant in this sensitivity analysis. This is to be expected since the binary variable construction sacrifices some information. But in neither cases did non-significant results become significant, nor was the direction of a significant effect reversed (see Supplemental Table S4).

## Discussion

### Summary of key findings

Patient durations in English EDs increased substantially between 2013 and 2018,^22^ and prolonged stays have been associated with worse patient outcomes.^23,24^ There were therefore potential concerns that NHS industrial action between 2022 and 2024 might further worsen ED flow.

We found that the effects varied by staff group. Resident doctor, consultant and paramedic strikes were associated with shorter ED durations, while nurse strikes showed little overall change. By contrast, simultaneous resident doctor and consultant strikes were associated with longer ED durations, indicating a qualitatively different operational context from strikes involving a single staff group. When only one medical group was on strike, opportunities for internal cover and service adaptation may have helped maintain flow. When both groups were absent, these options were likely more limited, making delays in senior decision-making, specialty review, admission authorisation and patient disposition harder to avoid. Longer durations during the combined strikes were also partly explained by higher walk-in demand, greater non-elective bed occupancy and more intensive testing or treatment. However, these measured factors explained only part of the observed effect, suggesting that unmeasured operational influences – such as staffing skill-mix, team coordination, leadership presence or local contingency arrangements – also played an important role.

Our analysis also suggests that changes in ED demand, hospital occupancy and clinical practice contributed to the shorter durations seen during other strike periods. In particular, patients were sometimes managed more intensively, admission thresholds appeared higher and efforts to meet the 4-h target increased, all of which were associated with shorter stays.

Substantial reductions in planned care activity, including elective admissions, outpatient activity and non-urgent diagnostic imaging, did not significantly improve ED flow. This suggests that measures directly affecting emergency demand, bed availability and front-line decision-making may be more important determinants of ED performance during periods of system pressure.

### Relationship with existing literature

Systematic review evidence shows that strikes are relatively common in healthcare across the world. They appear to have little or no impact on patient mortality^25^ and morbidity^26^ but do impact service delivery,^6^ although the mechanisms remain unclear.^27^

Several studies specifically analysed the impact of strike action in England. Ruiz et al.^4^ examined the impact of a 24-h doctors’ strike in English NHS hospitals on 21 June 2012. Compared to non-strike days, emergency admissions fell by 2.4%, while elective admissions dropped by 12.8%. Outpatient appointments saw a 7.8% decline in patients seen by medical staff, alongside a 45.5% increase in cancellations by hospitals. ED attendances also decreased by 4.7%. Furnivall et al.^4^ based on four resident doctor strikes in England in 2016 (January–April) also reported a 6.8% reduction in ED attendances, but did not focus on ED flow.

Ravioli et al.^28^ conducted a single-centre study examining two consecutive UK resident doctor strikes in March and April 2023. They reported improved ED patient flow during strike days, which they attributed primarily to increased senior staff presence. Garner et al.^29^ examined the impact of strike action during December 2022 and April 2024 in two EDs. They found that (across all staff groups – resident doctor strike, consultant strike, both resident doctor and consultant strike, nurse strike, ambulance strike) strike action was associated improved ED flow, likely attributable to increased capacity resulting from elective care postponements. Borchert et al.^30^ analysed three resident doctor strikes (March–June 2023) in a single radiology department. Imaging volumes fell temporarily by 5%–10%, mainly due to fewer X-ray and MRI studies, while urgent CT and MRI scans – particularly out-of-hours – were reported more quickly. This study is relevant to the ED because timely imaging and reporting are critical to emergency care, directly influencing diagnostic speed, patient flow and disposition decisions such as admission, discharge or escalation to specialist pathways. The preservation of urgent CT and MRI reporting during strike periods suggests that key emergency imaging services were prioritised, potentially helping to protect time-critical ED care and patient safety. However, this resilience depended on consultants covering registrar duties, which displaced non-clinical work such as teaching and research and led to fatigue during later strikes, raising concerns about whether this model is sustainable during prolonged or repeated industrial action.

A 2023 commentary summarised the reflections of consultants and senior staff who provided cover during the recent resident doctor strikes.^31^ The interviewed consultants highlighted the critical role that advanced clinical practitioners played, night-time availability of pharmacists and IT support and the deferment of non-clinical tasks. Many felt that the response to strike action had been a success, but the arrangements that were put in place were not sustainable. These insights highlight a number of issues that might explain the gap between the total effect of strikes on ED durations that we observed and mediated effects that we were able to assess.

Staff perspectives and team functioning may represent another important mechanism linking strike action to ED performance. In a recent (2026) qualitative study of UK health workers during the 2022–2024 NHS disputes, Essex and Smithard^32^ found that although strike periods were associated with increased stress and some interpersonal tension, they also generated solidarity, informal support networks and more adaptive ways of working. Participants described decisions being made ‘quicker and smoother’, with consultants and other staff reorganising to prioritise urgent care and support one another, particularly out of hours. These findings suggest that ED flow during industrial action may depend not only on staffing levels or attendance patterns but also on how effectively teams communicate, redistribute roles and maintain cohesion under pressure. However, the coexistence of improved efficiency with stress and conflict also suggests that these arrangements may be operationally effective without being sustainable. Further research should examine the contribution of teamwork and staff experience to strike-day ED performance.

Overall, our results align with earlier studies examining the contextual causes of ED delays and crowding, which highlight the influence of front door ED demand, boarding and inpatient occupancy (exit block).^33,34^

### Strengths and limitations

This study has several strengths, including a large, comprehensive dataset and the quasi-random timing of strikes, which strengthens causal inference. Given the observational nature of this study, we sought to carefully control for confounders, in line with our causal model, so as to adjust for factors other than industrial action and its consequences, that might have influenced patients’ ED durations. However, there are limitations. We were unable to locate information on the actual number and types of staff working in EDs during strikes, and so were unable to account for this explicitly in our models. Each mediator was analysed separately, as jointly modelling the full set of relationships between exposures, mediators, covariates and outcomes was not feasible, although sensitivity analyses suggested this had only a modest effect on results.

The mediation analysis also relies on the assumption of sequential ignorability: after adjusting for measured patient, temporal and provider factors, strike and non-strike attendances are assumed to be comparable, and there are no important unmeasured factors influencing both the mediator (e.g. bed occupancy or admissions) and ED duration. While the quasi-random timing of strikes supports this assumption, it cannot be guaranteed in an observational study. Residual confounding from unmeasured operational factors – such as staffing skill-mix, senior decision-maker availability, changes in patient handovers and team coordination, specialty response times, escalation processes or local contingency arrangements – may therefore remain. This may be particularly relevant to the longer ED durations observed during simultaneous resident doctor and consultant strikes. Potential changes in coding practices during strikes could also introduce bias.

Study sites were selected on data quality grounds, which may limit generalisability, although they appeared broadly representative of English Type 1 EDs. Some mediators were simplified for practicality: ED demand was grouped by arrival mode, bed occupancy was measured at the midpoint of the attendance and diagnostic tests were grouped into four categories. These choices may have obscured more granular effects.

Our analysis estimates average effects, which may vary by patient characteristics, timing or provider. Finally, we focused on ED flow; other outcomes such as reattendance, morbidity, mortality, post-strike system effects and impacts on staff well-being, teamwork and morale warrant further study.

### Implications for policy, practice and future research

Overall, strike days provided a valuable natural experiment that may help inform improvements in urgent and emergency care during future industrial action, other periods of system pressure and routine operations. Most strike periods were associated with shorter ED durations. However, simultaneous resident doctor and consultant strikes were associated with longer ED durations, making them an important exception. This was partly explained by higher ED demand, greater non-elective bed occupancy and some changes in testing intensity, but may also indicate that maintaining safe emergency cover is more difficult when both medical groups are absent. Delays in senior decision-making, specialty review, admission authorisation or patient disposition may have been harder to avoid under these conditions. Further research using richer operational data on staffing models, specialty support, leadership presence, teamwork and adaptive working practices may help explain the residual increase in ED duration not captured by our measured mediators.

During resident doctor strikes, consultants, other specialists, advanced clinical practitioners and senior nurses provided cover. Patients were managed more intensively and were less likely to be admitted, and ED durations fell modestly, suggesting that alternative staffing models and senior-led decision-making may help maintain flow while reducing pressure on inpatient beds. Hospitals also appeared to take pre-emptive steps to reduce inpatient occupancy before strikes, which was associated with shorter ED stays. Importantly, this did not appear to trigger compensatory increases in admissions through ED. This aligns with earlier evidence suggesting that inpatient occupancy influences ED admission thresholds mainly when occupancy reaches extreme levels.^35^

During the earlier nurse and paramedic strikes, ED demand fell and durations also shortened. This effect became less marked over time, possibly reflecting reduced public responsiveness as industrial action continued. Strategies aimed at reducing lower-acuity ED demand may therefore improve flow, but need to avoid discouraging patients who require urgent care. Changes in pre-hospital services, such as GP appointments, community contacts, and MIU activity, had comparatively modest effects.

Changes in diagnostic testing were associated with ED duration, but testing patterns during strikes were generally modest and inconsistent by strike type. It is unclear whether this reflected different clinical decision-making, changing test availability or recording practices.

Substantial reductions in planned care activity did not significantly improve ED flow, even on days of high demand or bed pressure. While reductions in elective work may have been necessary for staffing reasons, they do not appear to be an effective strategy for relieving pressure on EDs. This may support policies, such as surgical hubs, that seek greater separation between elective and emergency care pathways.^36^

The gap between the total and mediated effects of strike action also suggests that unmeasured mechanisms may be important, including staff morale, teamwork, adaptive working practices, specialty input, non-clinical responsibilities and training activity. These factors warrant further study.

## Conclusion

Industrial action – particularly during resident doctors and consultant strikes – was associated with reduced ED durations, due in part, to increased emergency bed availability, reduced ED demand, more intensive patient management and heightened focus on meeting performance targets. However, changes in ED flow during strikes could not be fully explained by the factors we assessed. This suggests that there may be unknown or under-reported mechanisms that impact ED flow. Notably, while reductions in planned care activities were substantial, they did not significantly impact ED flow.

## Supplemental Material

sj-docx-1-jrs-10.1177_01410768261461989 – Supplemental material for Industrial action and emergency department attendances in England, 2022–2024: a retrospective multicentre causal mediation analysis Supplemental material, sj-docx-1-jrs-10.1177_01410768261461989 for Industrial action and emergency department attendances in England, 2022–2024: a retrospective multicentre causal mediation analysis by Steven Wyatt, Mohammed A. Mohammed, Jonathan Clarke, Marta Garcia-Fiñana and Iain Buchan in Journal of the Royal Society of Medicine

## Declaration

**Ethical Approval:** Ethical approval was not required for this study. Patients and public were not involved in this study. The data and analyses that support the findings of this study are available on request from the corresponding author subject to prior approval by the data owner (NHS England).

**Guarantor:** SW

**Contributorship:** SW designed the study and undertook the analyses and drafted the first report. MAM provided critical comment and drafted the final manuscript. JC, MG-F and IB gave critical comments. All authors contributed to the final manuscript.

**Provenance:** Not commissioned; peer-reviewed by Julie Morris and Jack Greenwold.

**Use of Generative AI:** No generative AI was used during the preparation of this manuscript.
