# Supplementary material for: Industrial action and emergency department attendances in England, 2022–2024: a retrospective multicentre causal mediation analysis
Source: J R Soc Med. 2026 Aug 9;119(7):190–204. doi: 10.1177/01410768261461989 (PMC13457422; doi:10.1177/01410768261461989)
Supplement: Supplementary file 1 [file sj-docx-1-jrs-10.1177_01410768261461989.docx]

## Supplementary file

#### Note S1: Admission / discharge time imputation

Whilst the completeness of admission and discharge times in our sample was high (due to our data quality thresholds), we developed a kNN-based algorithm to impute times for (<1%) records without these values. This was necessary since missing admission or discharge times - for even a single patient - would invalidate a provider’s occupancy count indefinitely. Within provider, admission method, and day of the week groups, the kNN algorithm identified the record that was most similar (k =1) to the record with the missing value, based on a specified combination of admission time or discharge time (where valid), and length of stay. This combination was dependent on the pattern of missingness and whether the admission was a “same-day” admission. An additional random variable was added to the imputation process to avoid multiple selection from the same donor.

#### Figure S1: Derivation of study and model dataset

#### Table S1: Representativeness of study and model datasets

| grouping | group | All England | | Study dataset | | Model dataset | |
| --- | --- | --- | --- | --- | --- | --- | --- |
| all | all | 25,919,136 | 100% | 3,946,696 | 100% | 1,684,616 | 100% |
| sex | female | 13,307,340 | 51.3% | 2,032,116 | 51.5% | 867,549 | 51.5% |
|  | male | 12,564,163 | 48.5% | 1,913,640 | 48.5% | 817,067 | 48.5% |
|  | missing | 47,633 | 0.2% | 940 | 0.0% | 0 | 0.0% |
| age | 0 17 yrs | 6,101,910 | 23.5% | 919,398 | 23.3% | 401,498 | 23.8% |
|  | 18 - 64 yrs | 12,741,298 | 49.2% | 1,850,687 | 46.9% | 789,157 | 46.8% |
|  | 65+ yrs | 7,075,435 | 27.3% | 1,176,512 | 29.8% | 493,961 | 29.3% |
|  | missing | 493 | 0.0% | 99 | 0.0% | 0 | 0.0% |
| deprivation quintile | 1 most deprived | 6,764,989 | 26.1% | 942,530 | 23.9% | 395,580 | 23.5% |
|  | 2 | 5,688,403 | 21.9% | 844,185 | 21.4% | 365,342 | 21.7% |
|  | 3 | 4,997,011 | 19.3% | 751,637 | 19.0% | 324,215 | 19.2% |
|  | 4 | 4,507,230 | 17.4% | 717,131 | 18.2% | 307,082 | 18.2% |
|  | 5 | 3,959,013 | 15.3% | 690,993 | 17.5% | 292,397 | 17.4% |
|  | missing | 2,490 | 0.0% | 220 | 0.0% | 0 | 0.0% |
| arrival mode | by ambulance | 7,203,277 | 27.8% | 1,225,472 | 31.1% | 516,202 | 30.6% |
|  | walk in | 18,490,476 | 71.3% | 2,681,384 | 67.9% | 1,168,414 | 69.4% |
|  | missing | 225,383 | 0.9% | 39,840 | 1.0% | 0 | 0.0% |
| acuity | immediate | 350,178 | 1.4% | 60,127 | 1.5% | 24,448 | 1.5% |
|  | very urgent | 2,933,595 | 11.3% | 456,803 | 11.6% | 206,272 | 12.2% |
|  | urgent | 10,383,692 | 40.1% | 1,600,957 | 40.6% | 683,374 | 40.6% |
|  | standard | 9,602,046 | 37.0% | 1,664,643 | 42.2% | 730,472 | 43.4% |
|  | non-urgent | 765,390 | 3.0% | 92,704 | 2.3% | 40,050 | 2.4% |
|  | missing | 1,884,235 | 7.3% | 71,462 | 1.8% | 0 | 0.0% |
| duration in ED | < 1 hr | 1,663,450 | 6.4% | 243,171 | 6.2% | 97,445 | 5.8% |
|  | 1-3 hrs | 6,993,818 | 27.0% | 947,963 | 24.0% | 410,559 | 24.4% |
|  | 4-6 hr | 8,883,868 | 34.3% | 1,342,485 | 34.0% | 585,255 | 34.7% |
|  | 7+ hrs | 8,333,434 | 32.2% | 1,411,061 | 35.8% | 591,357 | 35.1% |
|  | missing | 44,566 | 0.2% | 2,016 | 0% | 0 | 0.0% |

#### Table S2: Impact of strikes on the odds of ED duration exceeding 4 and 12 hours

| **Strikes** | **4-hour breach** | | **12-hour breach** | |
| --- | --- | --- | --- | --- |
|  | **Odds ratio** | **(95% CI)** | **Odds ratio** | **(95% CI)** |
| Residents | 0.630 | (0.611 to 0.650) | 0.666 | (0.655 to 0.678) |
| Consultants | 0.798 | (0.731 to 0.871) | 0.870 | (0.829 to 0.912) |
| Residents + consultants | 1.156 | (1.026 to 1.301) | 1.280 | (1.189 to 1.377) |
| Paramedics | 0.880 | (0.826 to 0.938) | 0.821 | (0.788 to 0.856) |
| Nurses | 1.008 | (0.952 to 1.067) | 0.912 | (0.879 to 0.945) |

#### Figure S2: Mediated effect of industrial action on ED durations in minutes with 95% confidence intervals

#### Table S3: Sensitivity to violation of the sequential ignorability assumption

| **Exposure**  (Strike) | **Mediator** | **the value of rho at which average causal mediation is zero** | **the value of the product of *R square stars* for which the average causal mediation is zero** | **the value of the product of *R square tildes* for which the average causal mediation is zero** |
| --- | --- | --- | --- | --- |
| Residents | Non-elective bed occupancy | 0.1 | 0.010 | 0.006 |
| Consultants | Number of biochemistry tests | 0.2 | 0.040 | 0.021 |
| Residents + consultants | 4hr distortion effect | -0.1 | 0.010 | 0.007 |
| Paramedics | Number of ambulance-conveyed ED attendances | 0.0 | 0.000 | 0.000 |

#### Figure S3: Impact of allowing for causal interaction between selected mediator pairs

#### Average causal mediated effect on ED durations during resident doctor strikes

#### Table S4: Mediation results when treating ED testing mediators as binary (resident doctor strikes)

| Strike | Mediator | Impact of strike on mediator | | | Impact of mediator on ED Duration | | | % mediated | | |
| --- | --- | --- | --- | --- | --- | --- | --- | --- | --- | --- |
|  |  | Estimate | 95% CI | | Estimate | 95% CI | | Estimate | 95% CI | |
| Residents | yn imaging tests (patient) | 0.00 | -0.02 | 0.02 | 0.33 | 0.25 | 0.42 | 0% | -1% | 1% |
|  | yn blood tests (patient) | 0.00 | -0.02 | 0.01 | 0.53 | 0.42 | 0.65 | 0% | -1% | 1% |
|  | yn biochem tests (patient) | 0.00 | -0.02 | 0.02 | 0.56 | 0.44 | 0.68 | 0% | -1% | 1% |
|  | yn other tests (patient) | -0.01 | -0.03 | 0.01 | 0.20 | 0.13 | 0.26 | 0% | 0% | 1% |
| Consultants | yn imaging tests (patient) | 0.02 | -0.03 | 0.07 | 0.33 | 0.25 | 0.42 | -2% | -10% | 4% |
|  | yn blood tests (patient) | 0.08 | 0.03 | 0.14 | 0.53 | 0.42 | 0.65 | -10% | -28% | -4% |
|  | yn biochem tests (patient) | 0.11 | 0.05 | 0.16 | 0.56 | 0.44 | 0.68 | -12% | -23% | -5% |
|  | yn other tests (patient) | -0.02 | -0.07 | 0.03 | 0.20 | 0.13 | 0.26 | 2% | -2% | 5% |
| Residents+ consultants | yn imaging tests (patient) | 0.00 | -0.07 | 0.08 | 0.33 | 0.25 | 0.42 | 1% | -8% | 6% |
|  | yn blood tests (patient) | -0.02 | -0.10 | 0.06 | 0.53 | 0.42 | 0.65 | -2% | -12% | 6% |
|  | yn biochem tests (patient) | -0.02 | -0.11 | 0.06 | 0.56 | 0.44 | 0.68 | -2% | -12% | 5% |
|  | yn other tests (patient) | -0.05 | -0.12 | 0.02 | 0.20 | 0.13 | 0.26 | -3% | -9% | 1% |
| Paramedics | yn imaging tests (patient) | 0.00 | -0.04 | 0.04 | 0.33 | 0.25 | 0.42 | 0% | -6% | 6% |
|  | yn blood tests (patient) | 0.01 | -0.03 | 0.06 | 0.53 | 0.42 | 0.65 | -2% | -12% | 6% |
|  | yn biochem tests (patient) | -0.03 | -0.07 | 0.02 | 0.56 | 0.44 | 0.68 | 4% | -5% | 13% |
|  | yn other tests (patient) | 0.03 | -0.01 | 0.07 | 0.20 | 0.13 | 0.26 | -2% | -7% | 1% |
| Nurses | yn imaging tests (patient) | 0.07 | 0.03 | 0.10 | 0.33 | 0.25 | 0.42 | -19% | -667% | 155% |
|  | yn blood tests (patient) | 0.00 | -0.04 | 0.04 | 0.53 | 0.42 | 0.65 | 0% | -94% | 92% |
|  | yn biochem tests (patient) | -0.01 | -0.05 | 0.03 | 0.56 | 0.44 | 0.68 | 2% | -126% | 83% |
|  | yn other tests (patient) | 0.05 | 0.01 | 0.09 | 0.20 | 0.13 | 0.26 | -8% | -54% | 65% |

#### Figure S5: Simplified causal framework
